# Periclitoral Abscess Associated With Pubic Hair Grooming and Exercise

**DOI:** 10.7759/cureus.111209

**Published:** 2026-06-20

**Authors:** Tillie Schumann, Shannon Schellhammer, Alysse Alejandro-Hernandez, Melissa Schecther, Steve Carlan

**Affiliations:** 1 Obstetrics and Gynecology, Orlando Regional Medical Center, Orlando, USA

**Keywords:** abscess, high-intensity exercise, incision and drainage of abscess, infrared sauna, periclitoral

## Abstract

Periclitoral abscesses are uncommon occurrences that can develop spontaneously. In the United States, infrared-enhanced exercise equipment, when used with high-intensity aerobic workouts, is promoted for effective calorie burning and improved fitness. Among females, pubic grooming is widespread and can cause friction on the vulvar skin.

A 17-year-old female patient initially presented with vulvar swelling and pain. After a 10-day course of oral cephalexin failed, she presented to the ED and was found to have a 2.6-cm periclitoral abscess. She had a history of participating in infrared-heated sauna workout classes and routinely shaving her genital hair. She was admitted to the hospital; however, after two days of IV antibiotics and pain control, her symptoms had not resolved. The abscess became fluctuant, and incision and drainage were performed. At four-day follow-up, the wound was healing and the edema was resolving.

Preventing the initial abscess is important. A few cases have been reported in the literature, but no case has identified a risk associated with the combination of pubic grooming and high-intensity exercise using infrared-integrated equipment and sauna rooms. While temporal correlation does not imply causation, more information on periclitoral abscess formation is useful.

## Introduction

Periclitoral abscess is an uncommon infection in women, with few cases documented [1,2]. No standard treatment has been established [3]. A periclitoral abscess typically presents as a very tender, debilitating condition, often associated with vulvar swelling, dyspareunia, and dysuria, especially after failed oral antibiotic therapy [1,4].

It can occur spontaneously or in association with other conditions [2]. Risk factors include periclitoral pilonidal cyst tracts with trapped hair [5], local trauma from friction, genital mutilation, female circumcision, or hair follicle removal [4,6], and Crohn’s disease [7]. The frequency of progression from skin inoculation to abscess formation is unknown but is likely extremely rare. Periclitoral swelling in an adolescent patient can have several etiologies, including Bartholin gland pathology, paraurethral gland involvement, an infected epidermal inclusion cyst, folliculitis/furuncle, hidradenitis suppurativa, a pilonidal sinus tract, traumatic hematoma, sexually transmitted infection, or vulvar cellulitis [2].

For clinically stable, immunocompetent patients, oral antibiotics alone may be effective, but most fluctuant masses will either drain spontaneously or require incision and drainage. Notably, the recurrence rate is substantial, exceeding 70% in one report [2], and marsupialization may be necessary [3,4].

For about 10 years, workout classes have been offered in the U.S. in standalone, 120-degree Fahrenheit infrared-heated saunas designed for small groups of three. These spaces use exercise equipment with built-in infrared technology to maximize calorie burn and support fitness. The workout rooms operate 24/7 and feature virtual high-intensity interval training sessions. During workouts, heat, sweat, and friction are generated. Mechanical factors such as friction, heat, and skin maceration can increase the risk of skin infections [8]. It is unknown whether the combination of exercise-induced friction in infrared saunas and pubic grooming increases the risk of a periclitoral abscess.

We report a case involving a young woman with a periclitoral abscess temporally associated with pubic shaving and frequent, repetitive, intense physical activity in a hot sauna and on exercise equipment, including a treadmill and bicycle, with integrated infrared technology.

## Case presentation

A 17-year-old G0 presented with worsening vulvar swelling and pain over the past week. She reported regularly shaving her pubic hair with a razor and had shaved prior to the swelling but did not notice any cuts. She is active and regularly participates in a workout class in a 120-130°F infrared sauna, led by virtual instructors and divided into 30-minute isometric workouts and 15-minute high-intensity interval training sessions. She had exercised with this routine three to five times per week for approximately three months. She wore a properly fitted, moisture-wicking spandex workout suit. The patient also plays lacrosse year-round, a practice she has maintained for the past six years. Her usual workout routine is to attend her workout class, then shower and go to lacrosse, showering again within an hour of returning home. She had no significant past medical, family, or social history other than a history of a pilonidal abscess that spontaneously drained near the top of the cleft between her buttocks at age 15. She was not taking any routine prescribed medications. Her review of systems was limited to the groin. She denied back pain and had no chills or recorded fever. She had no recollection of recent direct vulvar traumatic injury. After a few days of vulvar swelling and pain, the patient visited her pediatrician, who noted a 3-cm erythematous swelling over the labia majora. The pediatrician prescribed cephalexin 500 mg orally three times daily (TID) for 10 days and mupirocin ointment. She was told to use warm perineal compresses. The patient complied but noted no improvement. The day after completing cephalexin, the patient presented to the ED for evaluation due to worsening vulvar pain and swelling. She denied vaginal bleeding and abnormal vaginal discharge. She denied fever and chills. The patient was not sexually active and had no history of sexually transmitted infections or vaginal infections. The patient reported regular menses occurring every 28 days with normal flow.

Upon her initial workup in the ED, her WBC count was 9,600 cells/µL, with a reference range of 4,500-11,000 cells/µL. Her vital signs were stable, and her BMI was 24.13. A vaginitis panel obtained via vaginal swab and chlamydia and gonorrhea testing obtained via urine were negative (Table 1).

**Table 1:** Laboratory values on admission.

Laboratory test	Test method/units	Normal range	Patient value
Vaginal swab: bacterial vaginosis	Transcription-mediated amplification (TMA)	Negative	Negative
Vaginal swab: vulvovaginal candidiasis	Transcription-mediated amplification (TMA)	Negative	Negative
Vaginal swab: trichomoniasis	Transcription-mediated amplification (TMA)	Negative	Negative
White blood cell count	Cells per microliter (cells/µL)	4,500-11,000	9,600
Urine *Chlamydia trachomatis*	Nucleic acid amplification test (NAAT)	Negative	Negative
Urine *Neisseria gonorrhoeae*	Nucleic acid amplification test (NAAT)	Negative	Negative

**Figure 1:**
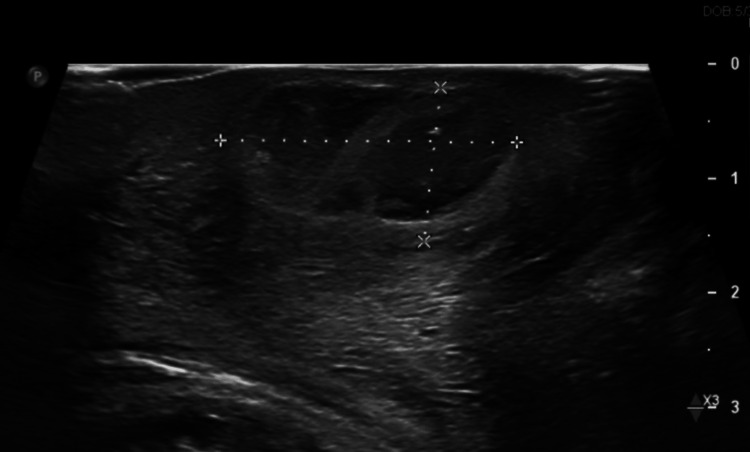
Translabial ultrasound showing a septated periclitoral abscess measuring 2.59 × 1.35 cm between cursors.

Translabial ultrasound was ordered and demonstrated a 2.6-cm complex fluid collection concerning for an abscess in the area of clitoral swelling (Figure 1).

The patient was subsequently admitted to the gynecology service. On initial physical examination, the clitoris was grossly enlarged with extensive induration. There was no significant erythema or visible cuts from recent shaving. A fluctuant pocket was not palpated. The swelling was very tender to palpation. The digital examination revealed no involvement of the vaginal canal. Inguinal nodes were negative. The patient was diagnosed with a clitoral abscess, and parenteral antibiotics were started on the recommendation of the infectious disease service. Intravenous doxycycline and metronidazole, 100 mg q12h and 500 mg q8h intravenous piggyback (IVPB), respectively, were given, along with ibuprofen 600 mg orally every eight hours for pain. The combination of antibiotics was chosen because of a high suspicion of a polymicrobial infection in a complex abscess. Anaerobes and community-acquired methicillin-resistant *Staphylococcus aureus* (MRSA) were targeted, along with broad coverage of atypical bacteria.

**Figure 2:**
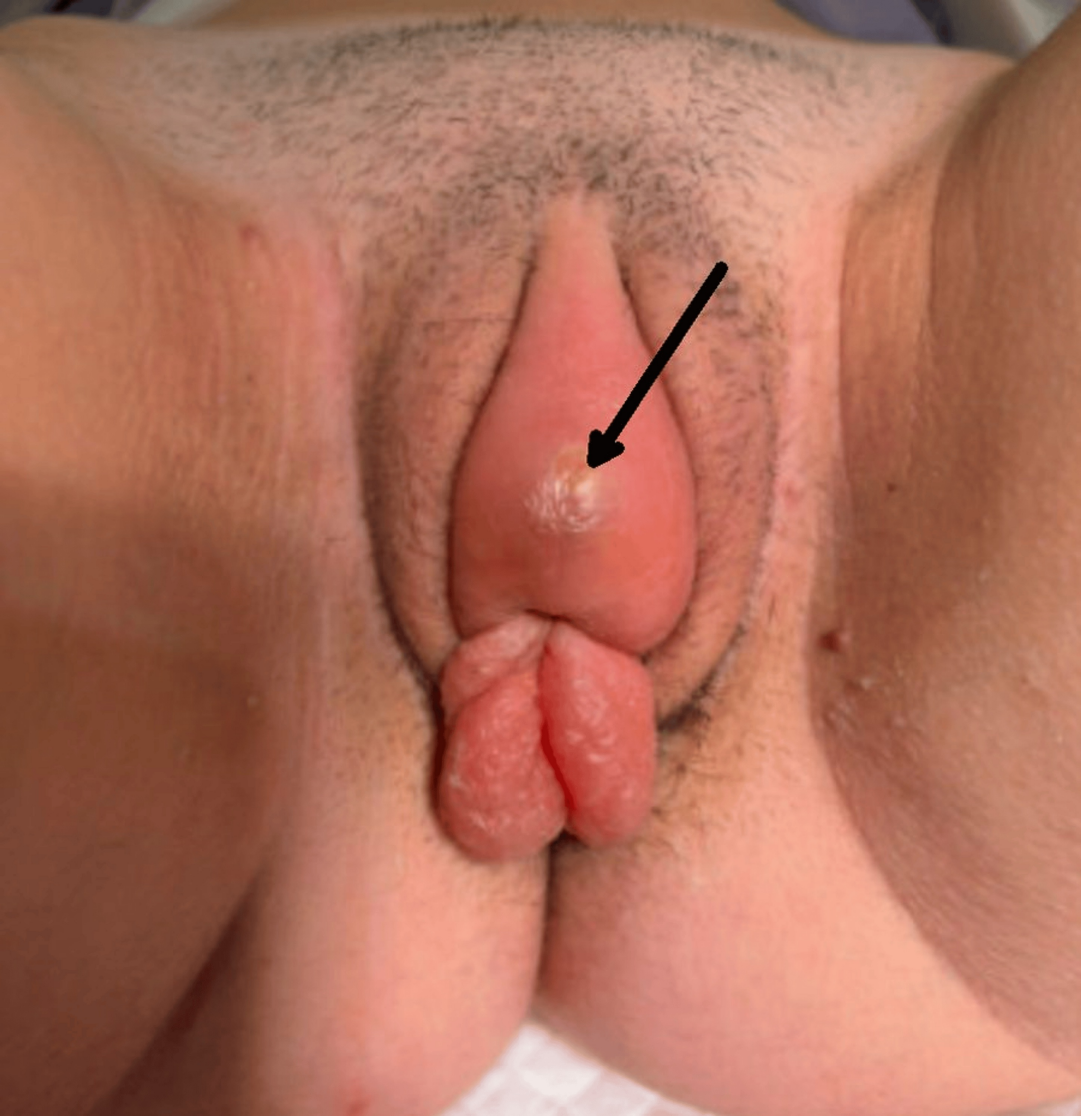
Image showing a grossly edematous and indurated abscess with fluctuance indicated by the arrow, taken immediately before incision and drainage in the operating room.

After 48 hours of antibiotics, the periclitoral area was grossly edematous and indurated. A central area of fluctuance was present (Figure 2).

She was taken to the operating room, and a 3-mm incision was made over the fluctuant head, yielding approximately 10 mL of purulent fluid for microbiologic studies. There was no evidence of paraurethral gland involvement. After incision and drainage, the wound was probed and found to track circumferentially, with no significant sinus tracts noted. The labia majora appeared normal; however, the labia minora were bilaterally edematous, without fluid or abscess extension into this area. The entire vulva was non-erythematous. The cavity was irrigated but not packed.

**Figure 3:**
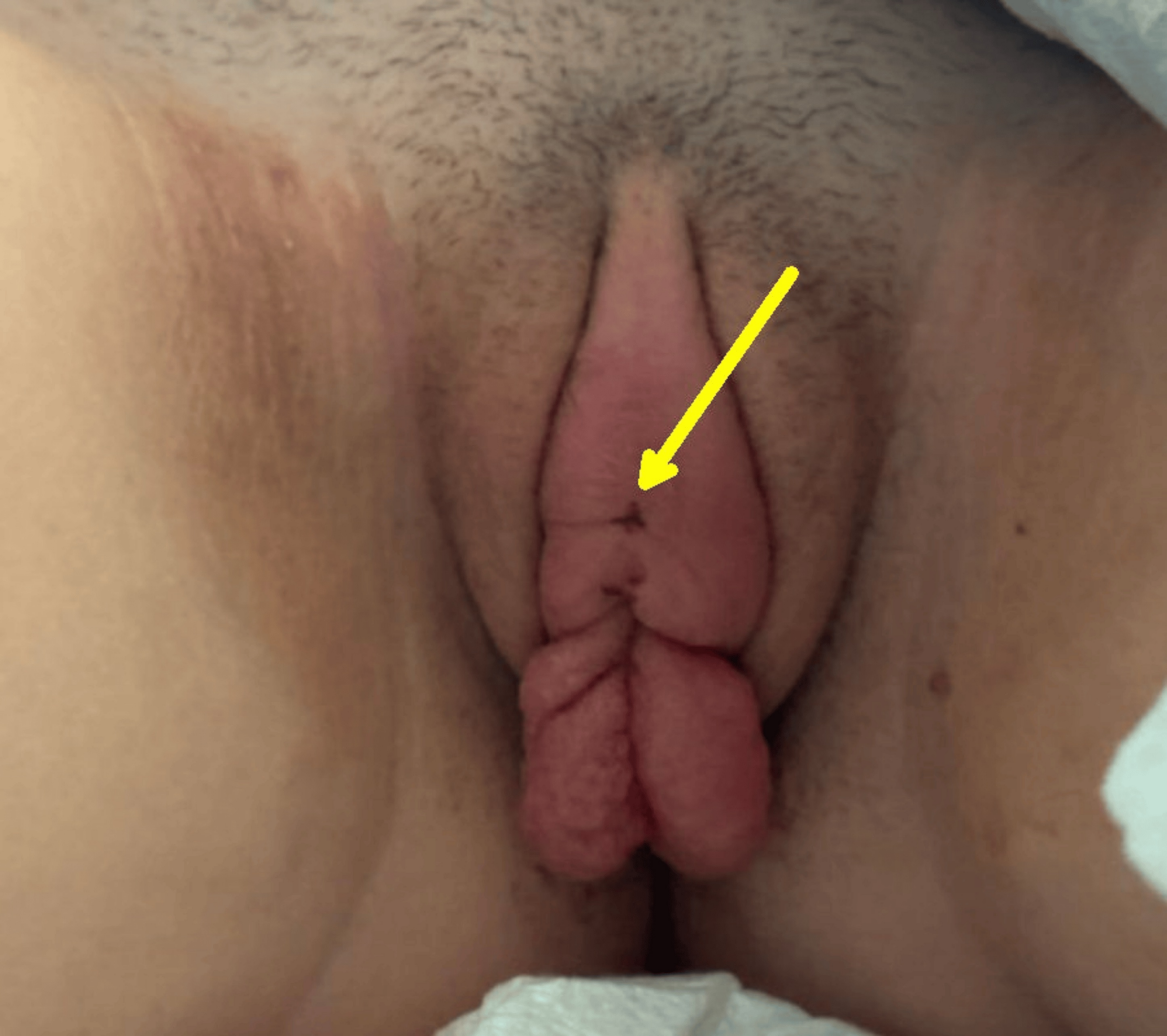
Image showing the healing infection, with the surgical drainage site indicated by the arrow.

She was discharged on postoperative day 1 with prescriptions for doxycycline 100 mg twice daily for seven days and metronidazole 500 mg twice daily for seven days. On the day of discharge, the clitoral wound was clean and dry, with no surrounding erythema or purulence. The labia and clitoral skin were mildly swollen but significantly improved from the previous examination. The patient followed up on postoperative day 4 in the outpatient clinic, and her pain had resolved. A physical examination in the clinic showed the vulva to be normal, with slight edema around the clitoral incision site (Figure 3).

The incision site was clean, dry, and intact, with no induration or loculations palpated and no erythema present. Final swab results showed an anaerobic culture with mixed microbial flora, with no predominant organism. Aerobic culture showed no growth, and Gram stain revealed numerous polymorphonuclear leukocytes, moderate numbers of Gram-positive cocci in pairs, and few Gram-negative bacilli. The patient completed the entire previously recommended antibiotic course. After her final outpatient visit, she returned to her previous activity and was lost to follow-up.

## Discussion

This case is important for three reasons. First, an abscess in the periclitoral area is a rare, painful condition, and this case adds to the available clinical information. Second, there is a significant risk of recurrence, underscoring the importance of identifying and mitigating antecedent risk factors. Third, interval high-intensity workouts using infrared-heated saunas with infrared-integrated equipment in women are a new development in the US over the last 10 years. There are reports of both exercise involving friction and perspiration in the groin [9] and pubic grooming [4,6] as possible risk factors, but the combination of infrared sauna exercise and pubic grooming has not been reported.

Fewer than 30 cases of periclitoral abscess have been reported in the English-language literature involving females aged 8 to 53 [10-11]. An optimal treatment strategy has not been defined, and the recurrence rate is high, resulting in potential long-term morbidity [3,4].

Regular exercise is a component of a healthy lifestyle and is widely recommended [12]. Although the overall incidence of exercise among American women is lower than among men, there is evidence of increasing participation in high-intensity interval aerobic training among women [13].

Full-spectrum infrared saunas became available to the public in the US in the late 1970s. The consumer literature reveals that they were commercialized for home use in the 1990s. Approximately 20 years ago, infrared technology was integrated into treadmills and other exercise equipment, but the technology remained largely in Europe. Nine years ago, stand-alone outlets began offering US consumers a 24-hour exercise space featuring an infrared sauna that reached 120-130°F, along with infrared-integrated exercise equipment, such as bicycles and treadmills, that heated the person rather than the ambient air [14].

Vigorous exercise in an environment of intense heat, combined with sauna-induced perspiration, can, if left undried, macerate the skin, promote overgrowth of commensal bacteria, and compromise the skin's integrity [15,16]. Only one previous report has examined the correlation between periclitoral abscess and aerobic exercise. We performed a MEDLINE search using the phrases “periclitoral abscess” AND “perspiration” AND “physical activity” and identified a case of a healthy 24-year-old woman with a periclitoral abscess associated with bicycle riding [9]. Unlike our case, this report did not involve infrared equipment.

Another notable variable is our patient’s habit of pubic grooming. Pubic shaving or waxing has been reported in association with periclitoral abscess and may be a risk factor [4]. Approximately 80% of surveyed women practice pubic grooming, with 50% having done so within the past 30 days [17]. A case from 2012 reported on a 17-year-old woman with a periclitoral abscess who had a history of pubic shaving. She had stopped shaving several weeks before presentation; consequently, the authors did not consider the shaving to be correlated with the abscess. There was no mention of exercise, athletics, heat, or sauna [2].

Strategies to avoid a periclitoral abscess and skin infection associated with high-intensity exercise in an infrared sauna environment and pubic grooming may include limiting infrared exposure time, controlling moisture, and exercising care during pubic grooming [18]. Keeping the area dry with powders, drying agents, or absorbents, and wearing loose-fitting, breathable natural fabrics may reduce moisture. Friction from pubic shaving and intense workouts may be mitigated with barrier creams [19,20]. Finally, hygiene, including showering immediately after exercise and changing clothes frequently, may reduce the conditions that lead to a skin infection and periclitoral abscess.

Compared with other case reports of periclitoral abscesses [4,16], our case was managed similarly, with conservative measures attempted first. Although no specific guidelines exist, many published reports follow similar management strategies. Conservative management may include observation, local heat application, and antibiotics [2]. Management then escalates to incision and drainage, and if the abscess recurs or does not resolve, the need for incision and excision or marsupialization is assessed [3]. Periclitoral abscesses that recur typically do so after one or more years [4].

Although the literature reports various organisms cultured from periclitoral abscesses, including coagulase-positive *Staphylococcus*, *Streptococcus bovis*, diphtheroids, and *Bacteroides*, our case did not yield a predominant pathogen in aerobic or anaerobic cultures. This could reflect the fact that she had been treated with antibiotics before cultures were obtained. The bacterial species does not appear to be an operative factor in whether a recurrence of the abscess occurs [4].

A limitation of this paper is that the temporal association between high-intensity exercise in an infrared sauna and pubic grooming alone does not imply causation of a periclitoral abscess. However, infrared exercise technology is new and gaining popularity. High-intensity interval training in an environment of intense heat, combined with sauna-room-produced perspiration that, if left undried, can macerate the skin and promote overgrowth of commensal bacteria [15], and friction from grooming on already irritated skin may be a risk factor for periclitoral abscess [16].

## Conclusions

Periclitoral abscesses can present spontaneously with marked vulvar pain and have high rates of recurrence regardless of initial management. Mitigating risk factors before an abscess develops in this sensitive region may decrease the risk of long-term complications. Separately, pubic grooming and bicycle exercise have been reported as possible antecedent risk factors. The current case reports high-intensity workout sessions in an infrared sauna on infrared-integrated equipment, combined with pubic grooming, as possible risk factors in a healthy 17-year-old.
